# Thioredoxin Reductase as a Novel and Efficient Plasma Biomarker for the Detection of Non-Small Cell Lung Cancer: a Large-scale, Multicenter study

**DOI:** 10.1038/s41598-018-38153-7

**Published:** 2019-02-25

**Authors:** Suofu Ye, Xiaofeng Chen, Yi Yao, Yueqin Li, Ruoxuan Sun, Huihui Zeng, Yongqian Shu, Hanwei Yin

**Affiliations:** 1 State Key Laboratory of Natural and Biomimetic Drugs, Peking University Health Science Center, Beijing, China; 2 Department of Oncology, The First Affiliated Hospital of Nanjing Medical University, Nanjing, China; 3 Cancer Center, Renmin Hospital of Wuhan University, Wuhan, China; 4 Keaise Center for Clinical Laboratory, Wuhan, China

## Abstract

There is an increased demand for efficient biomarkers for the diagnosis of non-small cell lung cancer (NSCLC). This study aimed to evaluate plasma levels of TrxR activity in a large population to confirm its validity and efficacy in NSCLC diagnosis. Blood samples were obtained from 1922 participants (638 cases of NSCLC, 555 cases of benign lung diseases (BLDs) and 729 sex- and age-matched healthy controls). The plasma levels of TrxR activity in patients with NSCLC (15.66 ± 11.44 U/ml) were significantly higher (P < 0.01) than in patients with BLDs (6.27 ± 3.78 U/ml) or healthy controls (2.05 ± 1.86 U/ml). The critical value of plasma TrxR activity levels for diagnosis of NSCLC was set at 10.18 U/ml, with a sensitivity of 71.6% and a specificity of 91.9%. The combination of NSE, CEA, CA19-9, Cyfra21-1, and TrxR was more effective for NSCLC diagnosis (sensitivity and specificity in the training set: 85.6%, 90.2%; validation set: 86.2%, 92.4%) than was each biomarker individually (P < 0.001). TrxR can also efficiently distinguish the metastatic status of the tumor, and it can further differentiate between various histological differentiations. Together, plasma TrxR activity was identified as a convenient, non-invasive, and efficient biomarker for the diagnosis of NSCLCs, particularly for discriminating between metastatic and non-metastatic tumors, or for histologic differentiation.

## Introduction

Currently, lung cancer is one of the most common malignancies and the most frequent cause of cancer-related death^1^. It is estimated that more than 1.8 million new lung cancer cases and around 1.6 million deaths associated with lung cancer occur annually worldwide, and these incidence and mortality rates are projected to undergo continued rapid growth^2^. Approximately 80% of lung cancer cases are categorized as being non-small cell lung cancer (NSCLC) based on histologic classification. Compared with other carcinomas, NSCLC carries a far worse prognosis and the overall 5-year survival rate after diagnosis remains below 15%^3–5^. Early and appropriate treatments for improving the 5-year survival rates largely depend upon the early and accurate diagnosis of lung cancer^6^. Unfortunately, delays commonly occur in the clinical diagnosis of lung cancers^7–10^. In China, the average diagnostic delay for lung cancer ranges from 2.9 to 8.4 months, and as a consequence 65.3% of the patients are first diagnosed at stage III or IV^11–15^.

At present, imaging techniques such as computed tomographic (CT) scans and chest X-rays play an important role in the clinical diagnosis of lung cancer. However, these tests have high false-positive rates and usually fail to uncover the hidden or subclinical lesions or small metastases, resulting in their limited application^16^. Furthermore, instances of over-diagnosis related to CT scans and the negative influences of radiation exposure have provoked widespread controversy. In addition, some invasive diagnostic strategies, including bronchoscopy and needle biopsy, are not widely utilized due to the associated pain and inconvenience^17^. Thus, the exploration of novel and efficient diagnostic biomarkers remains an urgent and necessary pursuit.

In recent years, a limited number of tumor-specific proteins have been identified, including neuron-specific enolase (NSE), carcinoembryonic antigen (CEA), cytokeratin fragment 21-1 (Cyfra21-1), and squamous cell carcinoma antigen (SCC-Ag). Some of these biomarkers, such as CEA and Cyfra21-1, have been applied in the clinical diagnosis of NSCLC according to the National Academy of Clinical Biochemistry guidelines^18^. However, the applications of these biomarkers remains unsatisfactory owing to the low sensitivity of diagnosis, which remains below 50%^19–21^. Hence, a top priority in lung cancer research should be the identification of a non-invasive, non-radiative, convenient, and fast diagnostic approach that offers both high sensitivity and specificity.

Thioredoxin reductase (TrxR) is a component of several redox-sensitive signaling cascades that mediate specific physiological processes, including those relating to cell survival, maturation, growth, migration, and inhibition of apoptosis^22–27^. This protein attracted our attention because it plays a key role in the tumor-related redox process^28^. Redox imbalance has long been recognized to be a factor in the pathology of NSCLCs^29,30^. Previous studies have found that TrxR is highly expressed in NSCLC both *in vitro* and *in vivo*, and is involved in regulating redox balance, transcription factor activities, and tumor growth in NSCLC^22,31,32^. In addition, the inhibition of TrxR activity by the TrxR-specific inhibitor Ethaselen resulted in a remarkable antitumor effect on the A549 NSCLC cell line as well as *in vivo* in NSCLC xenograft mice, suggesting that TrxR may play a crucial role in NSCLC^21,28^. It has also been reported that TrxR may be associated with aggressive tumor growth and poor patient outcomes^33–35^. More importantly for diagnostic purposes, the secretion of TrxR into the peripheral blood under conditions of oxidative stress has been observed and this secreted TrxR has proven to be a valuable indicator of oxidative stress^36–38^. These previous studies thus suggested that TrxR may be a potential and efficient biomarker for the diagnosis of NSCLC.

In our previous retrospective investigation, we evaluated a small cohort of patients with NSCLC (43 cases), revealing that plasma levels of TrxR activity were significantly higher in these patients than in healthy controls. In the present study, we further analyzed the diagnostic efficiency of TrxR activity as a plasma biomarker of NSCLC in a large population, and compared TrxR activity levels with different characteristics and features of the recruited patients and controls, including gender, age, body mass index (BMI), smoking history, histological type, tumor-node-metastasis (TNM) stage, metastasis, and histologic differentiation. Then, we further evaluated the levels of NSE, Cyfra21-1, CA19-9, CEA, TrxR and combinations thereof in a large clinical population to improve the efficiency of NSCLC diagnosis for future clinical application.

## Materials and Methods

### Patients

Patients with pathologically diagnosed NSCLC (638 cases), BLDs (555 cases), and sex- and age-matched healthy controls (729 cases), as shown in Supplemental Fig. S1, were continuously recruited from seven study centers, including Renmin Hospital of Wuhan University Cancer Center (Hubei, China), Peking University Third Hospital (Beijing, China), Zhejiang Cancer Hospital (Zhejiang, China), the First Affiliated Hospital of Nanjing Medical University (Jiangsu, China), Changyi People’s Hospital (Shandong, China), Hubei Traditional Chinese Medicine Hospital (Hubei, China) and Wuhan First hospital (Hubei, China), from 2011 to 2016. Patients with lung cancer (median age: 61 years; inter-quartile range: 41–80 years), including 224 cases of squamous cell carcinoma (SCC), 274 cases of adenocarcinoma (ADC), and 42 cases of large cell carcinoma (LCC), were initially diagnosed without undergoing antineoplastic therapy, radiotherapy, chemotherapy, surgery and other treatments. Patients diagnosed with BLDs (median age: 60 years; inter-quartile range: 36–82 years) were selected as a benign disease group, including 121 cases of lobar pneumonia, 196 cases of pulmonary TB with positive sputum cultures, 147 cases of chronic bronchitis, 41 cases of chronic obstructive pulmonary disease (COPD), and 50 cases of pulmonary inflammatory pseudotumor (PIP). All healthy controls (median age: 58 years; inter-quartile range: 40–72 years) were in normal conditions based on their complete blood test, liver/kidney functions and chest X-ray examination. NSCLC was defined on the basis of CT results and confirmed by histopathology according to the World Health Organization Classification of Tumors of the Lung^39^. Tumor stage was defined according to the 7th IASLC/AJCC staging system^40,41^. All samples were randomly separated into a training set and a validation set and matched with respect to age and sex. All patient clinicopathologic information, including age, gender, BMI, pathology, differentiation, TNM stage, and history of smoking is shown in Table 1.

**Table 1:** The characteristics of NSCLC, Benign lung disease, and healthy control cases in the training and validation cohorts.

Characteristics	Training Cohort			Validation Cohort		
	NSCLC	Benign lung disease	Healthy controls	NSCLC	Benign lung disease	Healthy controls
N	320	283	367	318	272	362
Age (IQR, years)	61 (41–79)	60 (36–81)	58 (40–71)	61 (40–81)	60 (32–84)	58 (41–72)
BMI (IQR, kg/m2)	25.4 (23.1–27.4)	25.7 (23.6–27.6)	25.7 (23.6–27.4)	25.5 (23.2–27.5)	25.6 (23.5–27.5)	25.7 (23.5–27.7)
**Gender (%)**						
Male	229 (71.56)	184 (65.02)	195 (53.13)	231 (72.64)	171 (62.87)	185 (51.1)
Female	91 (28.44)	99 (34.98)	172 (46.87)	87 (27.36)	101 (37.13)	177 (48.9)
**Lifetime smoking history (%**, **pack-years)**						
0	32 (10.00)	79 (27.92)	291 (79.29)	28 (8.81)	73 (26.84)	290 (80.11)
0–25	78 (24.38)	173 (61.13)	46 (12.53)	75 (23.58)	171 (62.87)	42 (11.60)
>25	210 (65.63)	31 (10.95)	30 (8.17)	215 (67.61)	28 (10.29)	30 (8.29)
**Histological type (%)**						
Squamous carcinoma	113 (42.01)	—	—	111 (40.96)	—	—
Adenocarcinoma	135 (50.19)	—	—	139 (51.29)	—	—
Large cell carcinoma	21 (7.81)	—	—	21 (7.75)	—	—
**TNM stage (%)**						
I	73 (22.81)	—	—	69 (21.7)	—	—
II	47 (14.69)	—	—	51 (16.04)	—	—
III	73 (22.81)	—	—	78 (24.53)	—	—
IV	127 (39.69)	—	—	120 (37.74)	—	—
**Distant metastasis (%)**						
No	193 (60.31)	—	—	198 (62.26)	—	—
Yes	127 (39.69)	—	—	120 (37.74)	—	—
**Histologic differentiation (%)**						
Well	27 (8.44)	—	—	33 (10.38)	—	—
Moderately	98 (30.63)	—	—	91 (28.62)	—	—
Poorly	144 (45.00)	—	—	147 (46.23)	—	—
Undifferentiated	51 (15.94)	—	—	47 (14.78)	—	—

### Specimen characteristics

5 ml samples of preoperative peripheral blood were collected in EDTA and anticoagulant-free tubes. Samples were centrifuged at 3,000 rpm at room temperature for 5 minutes within 2 hours of collection, and the supernatants were collected and tested immediately.

### TrxR activity assay

According to the previous literature^42^, both DTNB and Trx reduction assays were used to measure TrxR activity *in vitro*. For the DTNB reduction assay, the assessment of TrxR activity was based on the enzymatic activity of TrxR to catalyze the reduction of 5, 5′-dithiobis (2-nitrobenzoic) acid (DTNB) with NADPH to 5-thio-2-nitrobenzoic acid (TNB2-), which generates a strong yellow color with maximum absorbance at 412 nm^37,43^. For the Trx reduction assay, human recombinant Trx was added and incubated with mammalian TrxR, followed by monitoring the initial decrease in absorbance at 340 nm^37,43^. In biological samples such as human plasma, only DTNB reduction assay has been widely used to measure the TrxR activity^44–46^. All commercially available TrxR activity colorimetric assay kits, including BioVision (CA, USA), Sigma (MO, USA), and Clairvoyance (Wuhan, China), were designed to determine TrxR activity based on the DTNB reduction. Since several enzymes present in biological samples can reduce DTNB, a specific TrxR inhibitor is used to determine the reduction of DTNB due only to TrxR activity. However, the determination of TrxR activity in biological samples based on human recombinant Trx reduction assay has not been reported or validated in previous studies.

In this study, TrxR activity was measured by commercially available thioredoxin reductase (TrxR) activity colorimetric assay kits (BioVision, Milpitas, CA, USA and Clairvoyance Health Technology Co., Ltd, Wuhan, China), which was based on DTNB reduction and has been widely applied to determine the TrxR activity in human samples in many previous clinical studies^44–46^. Determination and calculation of TrxR activity were performed according to the manufacturer’s instruction and full details are provided in Supplemental Experimental Procedures. The coefficients of variation (CVs) of inter-assay and intra-assay were 4.2–8.1% and 5.9–8.8%, respectively. The detection range of this assay was between 0.5 U/mL-100 U/ml.

### Analysis of tumor markers

We examined four tumor markers [carcinoembryonic antigen (CEA), cancer antigen 19-9 (CA19-9, neuron-specific enolase (NSE), and cytokeratin 19 fragment (Cyfra21-1] in patients at the time of their first visit to the hospital. All patients gave their consent to having blood samples taken. CEA, CA19-9, NSE and Cyfra21-1 were assayed based on electrochemiluminescence immunoassay (ECLIA) and analyzed using a Cobas® analyzer (Roche Diagnostics, Mannheim, Germany). All reagents and kits were purchased from Roche diagnostics (Mannheim, Germany), and performed according to the manufacturer’s instruction. The upper normal limits for the tumor markers were 13 ng/ml for NSE, 5 ng/ml for CEA, 39 U/ml for CA19-9, and 3.3 ng/ml for Cyfra21-1.

### Statistical analysis

Results are described as percentages for categorical variables and as medians (interquartile ranges, IQRs) for the continuous variables. Proportions were calculated and compared using the Chi-squared test, while continuous variables between groups were compared by the non-parametric Mann-Whitney test with a Bonferroni correction. The diagnostic efficacy of biomarkers in NSCLC and BLD was evaluated based on the receiver operating characteristic (ROC) curves and the area under the curve (AUC) values. Correlations between plasma TrxR activity and confounding factors such as age, BMI and smoking history were analyzed primarily based on binary logistic regression analysis. Results are expressed as OR (odds ratio) values with the corresponding 95% CI (Confidence interval). All statistical analyses were performed using GraphPad Prism 6 (version 6.01; GraphPad, La Jolla, CA) and SPSS version 19.0 (SPSS Inc., Chicago, IL, USA). The significance threshold was set at 0.05 for two-tailed analyses.

### Ethics statement

This study protocol involving human participants was approved by the Ethics Committee of Renmin Hospital of Wuhan University Cancer Center (Hubei, China), Peking University Third Hospital (Beijing, China), Zhejiang Cancer Hospital (Zhejiang, China), the First Affiliated Hospital of Nanjing Medical University (Jiangsu, China), Changyi People’s Hospital (Shandong, China), Hubei Traditional Chinese Medicine Hospital (Hubei, China) and Wuhan first hospital (Hubei, China). The methods were carried out in accordance with the approved guidelines. Informed consent was obtained from all patients.

## Results

A total of 1922 participants were recruited, including 970 in the training cohort (320 patients with NSCLCs, 283 patients with BLDs and 367 healthy controls) and 952 in the validation cohort (318 patients with NSCLCs, 272 patients with BLDs and 362 healthy controls), as shown in Supplemental Fig. S1. The characteristics of the patients in training and validation cohorts are shown in Table 1. The training cohort and validation cohort were matched with respect to age and sex.

### The plasma levels of TrxR activity in patients with NSCLCs, patients with BLDs and healthy controls

**Figure 1:**
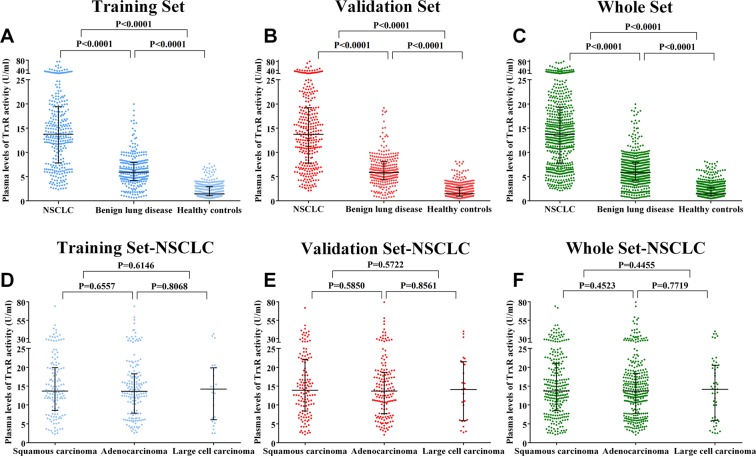
Scatter plot of the distribution of plasma TrxR activity levels. (**A**–**C**) The plasma levels of TrxR activity from individuals in the training (**A**), validation (**B**), and whole (**C**) sets. (**D–F**) The plasma levels of TrxR activity from patients with NSCLC in the training (**D**), validation (**E**), and whole (**F**) sets. The black horizontal lines are median values. P values were determined by the Mann–Whitney U test.

The plasma levels of TrxR activity (median ± IQR) in patients with NSCLCs, patients with BLDs, and healthy controls were detected and analyzed. In the training set, the plasma levels of TrxR activity in patients with NSCLCs (15.61 ± 11.63 U/ml) were significantly higher (P < 0.0001, Mann-Whitney U test, Fig. 1) than that in patients with BLDs (6.24 ± 3.75 U/ml) or healthy controls (2.04 ± 1.87 U/ml). In the validation set, the plasma levels of TrxR activity in patients with NSCLCs (15.66 ± 11.44 U/ml) were also significantly higher (P < 0.0001, Mann-Whitney U test, Fig. 1) than in patients with BLDs (6.27 ± 3.78 U/ml) or healthy controls (2.05 ± 1.86 U/ml). Similarly, the levels of TrxR activity in patients with NSCLCs was also significantly higher relative to patients with BLDs and healthy controls in the combined cohort.

Next, the correlation between plasma TrxR activity levels and NSCLC histological types was analyzed. In the training set, the median plasma TrxR activity levels in patients with SCCs, ADCs, and LCCs were 15.92 ± 11.34 U/ml, 15.51 ± 10.46 U/ml, and 14.55 ± 13.79 U/ml, respectively (Fig. 1). In the validation set, the median plasma TrxR activity levels in patients with SCCs, ADCs, and LCCs were 15.92 ± 11.34 U/ml, 15.42 ± 10.99 U/ml, and 15.07 ± 15.65 U/ml, respectively (Fig. 1). These differences in TrxR activity levels across different tumor histological types were not significant (P > 0.05) in either cohort. In addition, statistical analysis showed no influence of age or gender on TrxR activity levels in patients with NSCLCs (P > 0.05). Similar results were also obtained in the validation cohort.

### The efficacy of TrxR activity as a diagnostic biomarker of NSCLCs and BLDs

**Figure 2:**
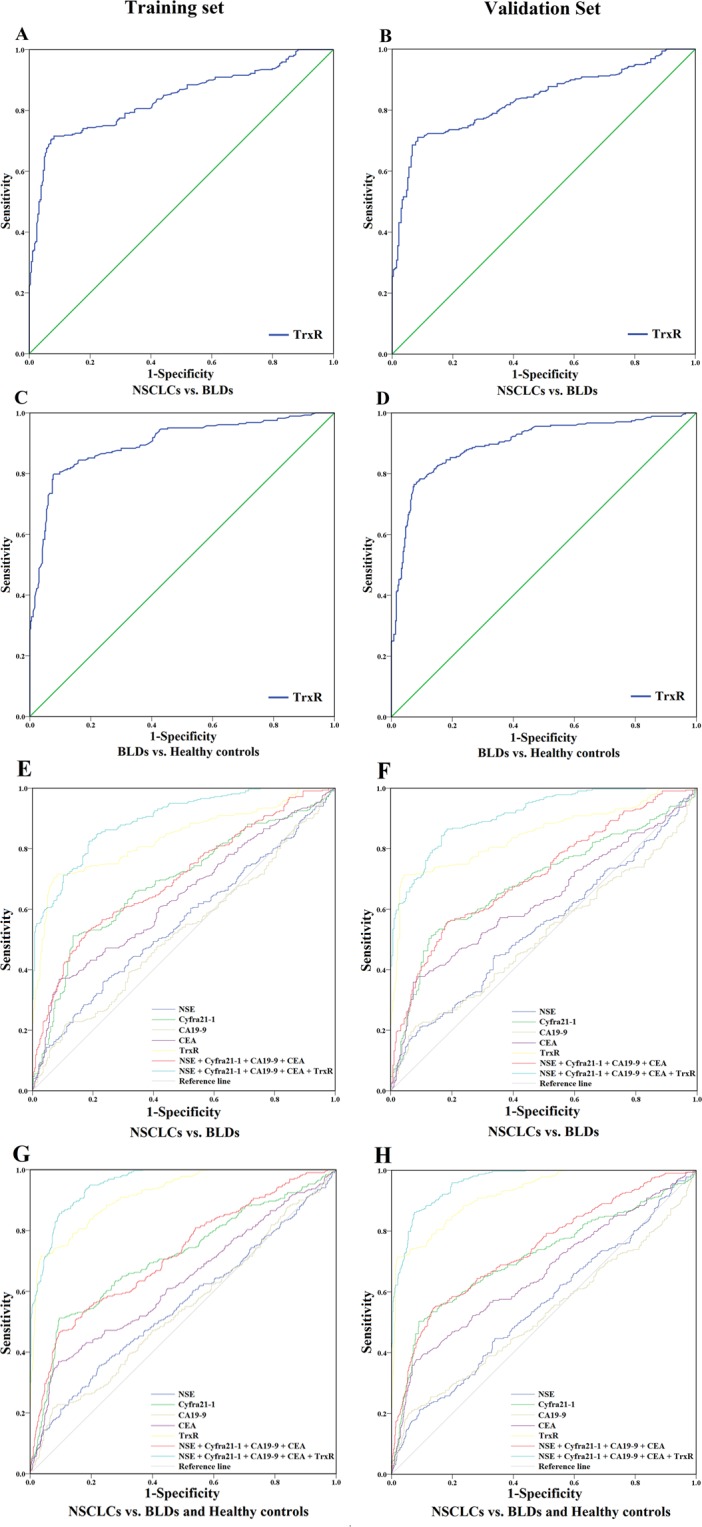
(**A**,**B**) ROC curve analyses of TrxR activity levels for the differentiation of NSCLC and BLD cases in the training (**A**) and validation (**B**) sets. (**C**,**D**) ROC curve analyses of TrxR activity levels for BLD cases vs. healthy controls in the training (**C**) and validation (**D**) sets. (**E**,**F**) ROC curve analyses of NSE, Cyfra21-1, CA19-9, CEA and TrxR and the combinations thereof for the differentiation of NSCLC and BLD cases in the training (**E**) and validation (**F**) sets. (**G**,**H**) ROC curve analyses of NSE, Cyfra21-1, CA19-9, CEA and TrxR and the combinations thereof for NSCLC patients vs. those with BLDs and healthy controls in the training (**G**) and validation (**H**) sets.

An analysis of ROC curves was performed in order to evaluate the efficacy of using TrxR activity as a biomarker for NSCLCs, with the maximal Youden Index (sensitivity + specificity-1) being used to determine the optimal cut-off value of TrxR activity as a means of distinguishing patients with NSCLCs from those with other conditions. Using this approach, the critical value of plasma TrxR activity levels for the diagnosis of NSCLC was set at 10.18 U/ml, based on the ROC curve (AUC 0.837; 95% CI, 0.805–0.869, Fig. 2) and the maximal Youden Index (0.634), with a sensitivity of 71.6% and a specificity of 91.9% (Table 2). In addition, TrxR activity levels also showed efficacy as a means of differentiating patient with BLDs from healthy controls. As shown in Fig. 2, the critical value of TrxR activity for diagnosis of BLDs from healthy controls was set as 4.01 U/ml, based on the ROC curve (AUC 0.899; 95% CI, 0.874–0.924) and the maximal Youden Index (0.720), with a sensitivity of 80.0% and a specificity of 92.1% (Table 2).

**Table 2:** The diagnostic efficiency of NSE, Cyfra21-1, CA19-9, CEA, TrxR and combinations thereof in differentiating between NSCLCs, BLDs, and healthy controls.

	Training Cohort							Validation Cohort						
	AUC (95% CI)	SEN(%)	SPE(%)	PPV(%)	NPV(%)	PLR	NLR	AUC (95% CI)	SEN(%)	SPE(%)	PPV(%)	NPV(%)	PLR	NLR
**NSCLCs vs**. **Benign lung diseases**														
NSE	0.554 (0.509–0.600)	21.30%	88.70%	68.07%	49.92%	1.88	0.89	0.538 (0.492–0.585)	21.40%	89.30%	70.04%	49.28%	2.00	0.88
Cyfra21-1	0.690 (0.647–0.732)	50.90%	86.60%	81.11%	60.93%	3.80	0.57	0.690 (0.647–0.733)	50.30%	87.50%	82.47%	60.09%	4.02	0.57
CA19-9	0.523 (0.477–0.569)	18.80%	90.10%	68.23%	49.53%	1.90	0.90	0.510 (0.463–0.557)	18.20%	93.70%	77.16%	49.49%	2.89	0.87
CEA	0.638 (0.594–0.682)	33.80%	91.90%	82.51%	55.11%	4.17	0.72	0.627 (0.582–0.672)	35.80%	92.30%	84.46%	55.15%	4.65	0.70
TrxR^a^	0.837 (0.805–0.869)	71.60%	91.90%	90.91%	74.11%	8.84	0.31	0.847 (0.815–0.879)	71.40%	95.60%	94.99%	74.09%	16.23	0.30
NSE + Cyfra21-1+ CA19-9 + CEA	0.702 (0.660–0.743)	52.50%	82.00%	76.73%	60.42%	2.92	0.58	0.711 (0.670–0.752)	55.70%	81.60%	77.97%	61.17%	3.03	0.54
NSE + Cyfra21-1+ CA19-9 + CEA + TrxR	0.897 (0.873–0.920)	82.50%	81.30%	83.30%	80.42%	4.41	0.22	0.912 (0.891–0.934)	86.50%	81.60%	84.61%	83.79%	4.70	0.17
**NSCLCs vs**. **Benign lung diseases and healthy controls**														
NSE	0.554 (0.513–0.594)	21.30%	89.80%	50.69%	69.86%	2.09	0.88	0.552 (0.512–0.592)	21.40%	90.40%	52.79%	69.63%	2.23	0.87
Cyfra21-1	0.712 (0.675–0.748)	50.90%	90.50%	72.51%	78.92%	5.36	0.54	0.713 (0.675–0.751)	50.30%	91.00%	73.71%	78.50%	5.59	0.55
CA19-9	0.539 (0.500–0.579)	18.80%	93.40%	58.37%	70.03%	2.85	0.87	0.522 (0.481–0.564)	18.20%	95.30%	66.01%	69.90%	3.87	0.86
CEA	0.628 (0.588–0.667)	33.80%	92.30%	68.36%	73.90%	4.39	0.72	0.657 (0.618–0.696)	35.80%	92.70%	71.10%	74.22%	4.90	0.69
TrxR^a^	0.919 (0.901–0.936)	71.60%	96.50%	90.97%	87.34%	20.46	0.29	0.923 (0.906–0.940)	71.40%	98.10%	94.96%	87.24%	37.58	0.29
NSE + Cyfra21-1+ CA19-9 + CEA	0.719 (0.684–0.755)	53.80%	84.20%	62.64%	78.73%	3.41	0.55	0.739 (0.704–0.774)	55.30%	85.50%	65.67%	79.22%	3.81	0.52
NSE + Cyfra21-1+ CA19-9 + CEA + TrxR	0.954 (0.943–0.966)	85.60%	90.20%	81.13%	92.71%	8.73	0.16	0.961 (0.951–0.971)	86.20%	92.40%	85.05%	93.03%	11.34	0.15
**NSCLCs vs**. **Benign lung diseases (The efficiency of TrxR in the diagnosis of NSE**, **Cyfra21-1**, **CA19-9**, **CEA -negative samples)**														
TrxR (NSE-negative samples)	0.831 (0.794–0.867)	63.89%	99.60%	99.38%	73.31%	160.36	0.36	0.828 (0.791–0.865)	64.00%	97.95%	96.97%	72.64%	31.23	0.37
TrxR (Cyfra21-1-negative samples)	0.729 (0.676–0.782)	42.41%	99.59%	98.53%	72.84%	103.89	0.58	0.728 (0.675–0.781)	44.30%	98.74%	95.89%	72.76%	35.15	0.56
TrxR (CA19-9-negative samples)	0.835 (0.800–0.871)	65.00%	99.61%	99.41%	73.62%	165.75	0.35	0.835 (0.799–0.870)	66.15%	98.43%	97.73%	73.96%	42.01	0.34
TrxR (CEA-negative samples)	0.796 (0.754–0.839)	57.08%	99.23%	98.37%	73.93%	74.20	0.43	0.789 (0.746–0.833)	55.39%	97.61%	94.96%	72.92%	23.17	0.46
**NSCLCs vs**. **Benign lung diseases and healthy controls (The efficiency of TrxR in the diagnosis of NSE**, **Cyfra21-1**, **CA19-9**, **CEA -negative samples)**														
TrxR (NSE-negative samples)	0.922 (0.903–0.940)	64.68%	98.97%	96.45%	86.66%	62.96	0.36	0.921 (0.902–0.939)	64.00%	99.10%	96.87%	86.34%	71.11	0.36
TrxR (Cyfra21-1-negative samples)	0.876 (0.848–0.903)	45.57%	99.66%	97.30%	87.20%	133.97	0.55	0.875 (0.848–0.903)	44.30%	99.50%	96.04%	86.71%	88.60	0.56
TrxR (CA19-9-negative samples)	0.922 (0.904–0.941)	65.00%	99.18%	97.13%	86.87%	78.91	0.35	0.921 (0.902–0.940)	66.15%	99.34%	97.73%	87.19%	99.73	0.34
TrxR (CEA-negative samples)	0.904 (0.882–0.926)	58.02%	99.17%	96.09%	86.99%	69.62	0.42	0.901 (0.878–0.924)	55.40%	99.00%	95.05%	86.48%	55.40	0.45

Abbreviations: SEN: sensitivity; SPE: specificity; PPV: positive predictive value; NPV: negative predictive value; PLR: positive likelihood ratio; NLR: negative likelihood ratio.

^a^The diagnostic cut-off value of TrxR activity levels was 10.18 U/ml.

### The diagnostic value of TrxR activity levels in NSCLC diagnosis in the validation cohort

By applying the optimal cut-off value of TrxR activity for diagnosing NSCLCs to our validation cohort, we were able to further confirm the effectiveness and efficiency, as the positive predictive value (PPV) of TrxR activity levels (>10.18 U/ml) was 94.99% for NSCLC diagnosis. Furthermore, in the assessment of our validation cohort, TrxR activity also showed great AUC 0.847 (95% CI, 0.815–0.879, Fig. 2) with a sensitivity of 71.40% and a specificity of 95.60% for the auxiliary diagnosis of NSCLCs, which was very similar to the training cohort. Also, based on the ROC curve, the critical TrxR activity value (>4.01 U/ml) for the diagnosis of BLDs (AUC 0.899; 95% CI, 0.873–0.925) yielded a sensitivity of 78.31% and a specificity of 90.60%. More importantly, only 22 patients with BLDs (8.02%) had a plasma TrxR activity level >10.18 U/ml. These results consistently validated the diagnostic efficacy of TrxR activity levels.

### ROC analyses and comparisons of NSE, Cyfra21-1, CA19-9, CEA, TrxR and combinations thereof for NSCLC diagnosis

The diagnostic efficiencies of TrxR activity, as well as those of the existing NSCLC clinical biomarkers NSE, Cyfra21-1, CA19-9, and CEA were evaluated and compared as a means of differentiating between patients with NSCLCs, BLDs, and healthy controls. The sensitivity, specificity, predictive value, and likelihood ratios of each marker are summarized in Table 2.

Among the five NSCLC biomarkers, TrxR activity displayed the highest AUC (Fig. 2 and Table 2; training set: 0.919; 95% CI, 0.901–0.936; validation set: 0.923; 95% CI, 0.906–0.940) and effectively distinguished NSCLCs from BLDs and healthy controls. Cyfra21-1 displayed the second highest AUC in differentiating patients with NSCLCs from other groups (Fig. 2 and Table 2; training set: 0.712; 95% CI, 0.675–0.748; validation set: 0.713; 95% CI, 0.675–0.751). CEA and NSE exhibited moderate capacities for differentiating NSCLCs from other groups with AUCs of 0.628 (95% CI, 0.588–0.667) and 0.554 (95% CI, 0.513–0.594), respectively. CA19-9 exhibited a low discriminatory capacity for NSCLCs with an AUC ranging from 0.500–0.579. These results showed that the diagnostic efficacy of TrxR in NSCLCs was greater than that of NSE, CEA, CA19-9, Cyfra21-1 (P < 0.001, Table 2).

Through a binary logistic regression, the combination of NSE, CEA, CA19-9, and Cyfra21-1 was found to exhibit an improved diagnostic efficiency for NSCLCs (training set: AUC, 0.719; 95% CI, 0.684–0.755; validation set: AUC 0.739; 95% CI, 0.704–0.774) relative to any individual biomarker (P < 0.001, Table 2 and Fig. 2). In addition, when adding TrxR into this combined group, diagnostic efficacy was further strengthened (training set: AUC, 0.954; 95% CI, 0.943–0.966; validation set: AUC 0.961; 95% CI, 0.951–0.971; Table 2) for NSCLCs relative to TrxR alone or to the combination of the other four biomarkers (P < 0.001, Table 2 and Fig. 2). These results offered an excellent diagnostic modality for NSCLCs suitable for clinical applications.

Furthermore, TrxR activity maintained diagnostic accuracy for patients with NSCLCs who were NSE-negative (Fig. 2 and Table 2; training set: 0.831; 95% CI, 0.794–0.867, sensitivity 63.89%, specificity 99.60%; validation set: 0.828; 95% CI, 0.791–0.865, sensitivity 64.00%, specificity 97.95%), Cyfra21-1-negative (training set: 0.729; 95% CI, 0.676–0.782, sensitivity 42.41%, specificity 99.59%; validation set: 0.728; 95% CI, 0.675–0.781, sensitivity 44.30%, specificity 98.74%), CA19-9-negative (training set: 0.835; 95% CI, 0.800–0.871, sensitivity 65.00%, specificity 99.61%; validation set: 0.835; 95% CI, 0.799–0.870, sensitivity 66.15%, specificity 98.43%) and CEA-negative (training set: 0.796; 95% CI, 0.754–0.839, sensitivity 57.08%, specificity 99.23%; validation set: 0.789; 95% CI, 0.746–0.833, sensitivity 55.39%, specificity 97.61%). These findings also verified the efficacy of TrxR activity as a means of diagnosing cancer in a high-risk population, such as populations in which the recommended routine screening is negative.

### The relationship between the severity of lung diseases and smoking status

Chi-squared tests were conducted to explore the relationship between smoking status and severity of lung diseases (healthy, benign, and malignant), revealing that smoking status significantly correlated with the severity of lung diseases (P < 0.001). Also, according to linear-by-linear association results, the severity of lung diseases was elevated as the pack-years of lifetime smoking history increased in a linear relationship (P < 0.001). Pearson correlation analysis indicated no correlation between TrxR activity levels and smoking history (pack-years) in any of the groups. We also conducted an ROC analysis in our study according to smoking history and the AUCs of the ROC curves were similar for smokers and non-smokers.

### The relationship between TrxR activity levels, metastasis, and TNM stage

**Figure 3:**
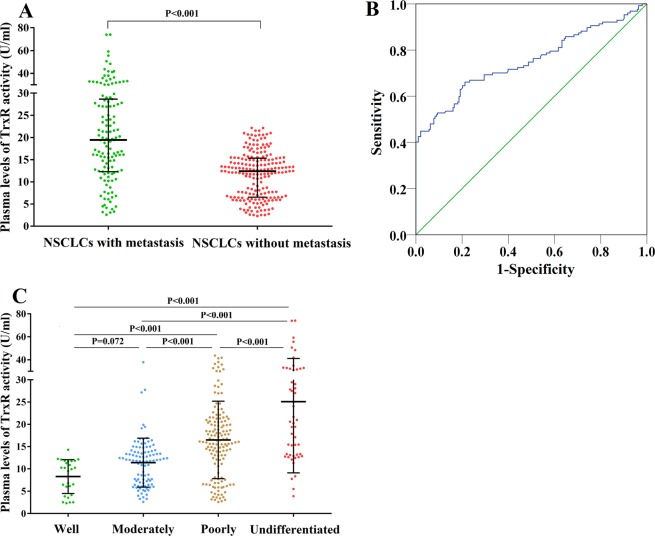
(**A**) Scatter plot of the distribution of plasma TrxR activity levels in metastatic and non-metastatic NSCLC. (**B**) ROC curve analyses of TrxR activity levels for the differentiation of metastatic NSCLC from non-metastatic NSCLC. (**C**) The plasma levels of TrxR activity of NSCLC with various histologic differentiations.

In the training cohort, the plasma levels of TrxR activity in the 127 NSCLC patients with metastatic tumors (median 19.45 U/ml; IQR, 16.35 U/ml) were significantly higher (P < 0.001) than in those patients with non-metastatic NSCLCs (12.39 U/ml; IQR, 8.80 U/ml), whereas no other markers were able to effectively distinguish metastasis (P > 0.05, Fig. 3). These results were further confirmed in our validation cohort. Furthermore, by conducting an ROC analysis, the efficacy of TrxR activity in the diagnosis of metastasis was investigated (AUC 0.751; 95% CI, 0.692–0.810, Fig. 3) and the optimal cut-off value for distinguishing metastasis was set at 16.00 U/ml with a sensitivity of 66.14% and a specificity of 78.76%. Based on the logistic regression, the TrxR activity remained an independent metastasis predictor with an adjusted OR of 1.269 (95% CI, 1.183–1.362), as compared with age, gender, BMI, history of smoking, NSE, Cyfra21–1, CA19-9, CEA, and histologic differentiation. In addition, histologic differentiation was also significantly associated with metastasis. TrxR activity levels in NSCLCs patients with different TNM stages were also investigated and no significant differences (P > 0.05) in TrxR activity levels were detected among those with TNM I, II, and III stages. However, the plasma TrxR activity levels in NSCLC patients of TNM IV were significantly higher (P < 0.001) than that in those patients of other TNM stages.

### The relationship between TrxR activity levels and histologic differentiation

The undifferentiated NSCLC patients had the highest median plasma level of TrxR activity at 20.60 U/ml (IQR, 19.17 U/ml), while those with poorly-differentiated NSCLCs ranked second (Median 16.10 U/ml; IQR, 10.01 U/ml; Table 3 and Fig. 3). The median TrxR activity level of well-differentiated NSCLC patients was the lowest (Median 11.94 U/ml; IQR, 7.10 U/ml; Table 3). Bonferroni corrections were also conducted to investigate the differences among histologic differentiation statuses, revealing that each differentiation status differed significantly (P < 0.01) from each other with the exception of well-differentiated and moderately-differentiated (P = 0.720). Other biomarkers, such as NSE, Cyfra21-1, CA19-9, and CEA, were also testified for their association with histologic differentiation, showing no ability to differentiate based on histologic differentiation (P > 0.05).

**Table 3:** The critical values of TrxR activity for diagnosing different diseases and differentiating based on histologic differentiation status.

	lower limit		upper limit	Diagnostic efficiency		
				sensitivity	specificity	AUC
**The optimal cut-off value of Plasma TrxR activity levels for distinguishing different disease backgrounds**						
Healthy	0.50 U/ml^a^		4.01 U/ml	—	—	—
Benign lung diseases	4.01 U/ml		10.18 U/ml	80.0%	92.1%	0.899
NSCLCs without metastasis	10.18 U/ml		16.00 U/ml	71.6%	91.9%	0.837
NSCLCs with metastasis	16.00 U/ml		100.00 U/ml^a^	66.14%	78.76%	0.751
**Plasma TrxR activity levels in NCSLSs with different histologic differentiations**						
	**N**	**Median**	**IQR**	**Bonferroni correction**		
				**P-value**		
Well	27	9.93	7.60	P = 0.720 (vs.Moderately)	P < 0.001 (vs.Poorly)	P < 0.001 (vs.Undifferentiated)
Moderately	98	11.94	7.10	P = 0.720 (vs.Poorly)	P < 0.001 (vs.Well)	P < 0.001 (vs.Undifferentiated)
Poorly	144	16.10	10.01	P < 0.001 (vs.Well)	P < 0.001 (vs.Moderately)	P < 0.001 (vs.Undifferentiated)
Undifferentiated	51	20.60	19.17	P < 0.001 (vs.Poorly)	P < 0.001 (vs.Moderately)	P < 0.001 (vs.Well)

^a^0.50 U/ml and 100.00 U/ml are the lower and upper limits of the linear range of TrxR activity detection assay.

## Discussion

TrxR plays a crucial role in the tumor-associated redox process which is closely related to the pathology of cancer^28–30^. In fact, overexpression of TrxR has been observed in multiple types of tumor according to previous literature and NCI-60 screening, a panel of 60 human cancer cell lines used by NCI to detect potential anticancer activity^22,32,33,47–49^. Among the tumor types with the highest TrxR expression, NSCLC was found to possess a significant and robust elevation of TrxR activity, which was further confirmed both *in vitro* and *in vivo*^22,32,33^. The upregulation of TrxR in NSCLC was demonstrated to be involved in regulating redox balance, transcription factor activities, and tumor growth in non-small cell lung carcinoma^31^. These findings provided a rationale for suggesting that TrxR may be a potential biomarker for the diagnosis of NSCLC. Additionally, TrxR is secreted into the blood under conditions of oxidative stress, inflammation, and in certain some other exceptional circumstances, making TrxR activity a relatively non-invasive and easily-detected potential plasma biomarker^36–38^.

In this study, the detection of plasma TrxR activity levels in 320 patients with NSCLCs, 283 patients with BLDs and 367 healthy controls in a training cohort was conducted, revealing that TrxR activity levels in patients with BLDs (6.24 ± 3.75 U/ml) were significantly higher than in healthy controls (2.04 ± 1.87 U/ml), which may be the result of the cell damage associated with lobar pneumonia, pulmonary TB, chronic bronchitis, COPD, and PIP^50–52^. More importantly, the TrxR activity levels in NSCLCs (15.61 ± 11.63 U/ml) were further elevated and significantly different from that in benign diseases, potentially due to increased oxidative stress and extensive TrxR secretion under tumor growth conditions^28–30,34,37,38^. Although there were no differences in activity levels among the three histological types of NSCLCs, partly because of the similar oxidative status of SCCs, ADCs, and LCCs, TrxR activity could effectively and efficiently distinguish these patients from BLDs and healthy controls, making up for the absence of an effective biomarker for diagnosing ADCs and LCCs. In addition, very similar results and identical conclusions were drawn from our validation cohort, including 318 patients with NSCLCs, 272 patients with BLDs, and 362 healthy controls, thus verifying the effectiveness of plasma TrxR activity levels as a means of discriminating among NSCLCs, BLDs and healthy controls.

Importantly, an appropriate sample preparation procedure is essential for the accurate determination of TrxR activity levels in plasma. In this study, we excluded all samples with evidence of hemolysis and lipemia as hemolysis and lipemia have both been shown to skew results. The effect of hemolysis is likely due to additional TrxR release from red blood cells, while lipemia has been shown to lead to a significant interference with the reaction system itself. The construction of reliable diagnostic criteria for TrxR activity levels capable of discriminating between NSCLCs and BLDs is important for future clinical application. By conducting ROC analyses and establishing AUCs for TrxR activity, we confirmed its diagnostic efficiency in discriminating between BLDs and NSCLCs with AUCs of 0.899 (95% CI, 0.874–0.924) and 0.837 (95% CI, 0.805–0.869), respectively. In addition, we set 4.01 U/ml and 10.18 U/ml as the critical cut-off values for the diagnosis of BLDs and NSCLCs, respectively, based on the maximal Youden Indexes, which appropriately balanced the sensitivity and specificity. Thus, the diagnostic criteria for NSCLCs and BLDs were constructed as shown in Table 3.

Some biomarkers have previously been used for the clinical diagnosis of NSCLCs, such as Cyfra21-1, NSE, and CEA. Cyfra21-1 has exhibited a good diagnostic capability in patients with SCCs but an unsatisfactory diagnostic efficacy in those with ADCs^17^, which may result in the limitation of its clinical application in the diagnosis of NSCLCs (sensitivity 50.90%, specificity 90.50%). CEA and NSE have been applied as biomarkers to the diagnosis and prognosis of several cancer types, including as a means of monitoring recurrence and evaluating prognosis in NSCLCs^53–57^, but these markers only performed moderately well as a means to diagnose NSCLCs in our training cohort (sensitivity 33.80%, specificity 92.30%; sensitivity 21.30%, specificity 89.80%, respectively). In the present study, we also incorporated the biomarker CA19-9, which has mainly been used for the diagnosis of several types of gastrointestinal cancers^58^, but found that its sensitivity only reached 18.8%. Compared to these individual biomarkers, TrxR activity appeared to be more effective and efficient for NSCLC diagnosis, with a sensitivity of 71.60% and a specificity of 96.50%. We further established a joint detection model for the diagnosis of NSCLCs, incorporating Cyfra21-1, NSE, CEA, CA19-9, and TrxR activity in our panel. The combination of Cyfra21-1, NSE, CEA and CA19-9 improved diagnostic sensitivity to 53.8% for NSCLCs. After adding TrxR activity into this diagnostic panel, the sensitivity greatly increased to 85.6%. In addition, TrxR activity maintained great diagnostic accuracy for patients with NSCLCs who were negative for recommended routine biomarker screening (NSE, Cyfra21-1, CA19-9, and CEA). These outcomes were further verified in our validation cohort (Table 2 and Fig. 2). Through analyses of a large cohort of 638 NSCLC patients, 555 BLD patients and 729 healthy controls, TrxR activity was thus well validated as a highly effective and efficient diagnostic marker for NSCLCs.

This study also revealed that NSCLC patients had on average a significantly longer smoking history than the healthy controls. Chi-squared tests and linear-by-linear association results indicated that the severity of lung diseases (from benign to malignant) elevated as the pack-years of lifetime smoking history increased, with a linear relationship between these two factors (P < 0.001). According to a Pearson correlation analysis results, there is no correlation between TrxR activity levels and smoking history (pack-years) in any of the assessed groups.

We also investigated correlations of TrxR activity and various clinical characteristics and features of patients with NSCLCs, such as metastasis, TNM stage, and histologic differentiation. Notably, previous studies have shown that TrxR plays a key role in mediating tumor metastasis^45,59,60^. In this study, the level of TrxR activity also increased significantly as NSCLCs progressed to a metastatic state. An ROC analysis indicated that the optimal cut-off value for distinguishing between metastatic and non-metastatic patients was 16.00 U/ml, with a sensitivity of 66.14% and a specificity of 78.76%, as shown in Table 3, whereas other biomarkers appeared to be of no value for diagnosing metastasis. We also investigated the correlation between TrxR activity and TNM stages, determining that no significant differences in TrxR activity levels were present among TNM I, II, and III patients. However, plasma TrxR activity levels in NSCLC patients of TNM IV were significantly higher (P < 0.001) than those of other TNM stages, presumably because TNM IV represented those patients with metastatic tumors. In addition, a Bonferroni correction showed that the TrxR activity levels tended to be higher as NSCLC patient tumors became less well-differentiated, while other biomarkers did not differ based on this histological criterion. This may be partly attributed to the fact that less differentiated NSCLCs tend to have higher degrees of malignancy, which can be quantitatively evaluated based on TrxR activity levels^45^.

Several previous studies have suggested TrxR as a potential biomarker in NSCLCs^46,61^. For example, Chen *et al*. conducted a restrospective clinical study and identified TrxR as an independent poor prognostic indicator for EGFR wild type and ALK negative NSCLC patients^46^. Another study performed by Zhou *et al*. found that almost 90% of NSCLCs patients (138/149) have shown a significant decrease of TrxR level after surgery, suggesting TrxR activity as a promising biomarker during NSCLC surgery and clinical treatment^61^. In the current research, we mostly focused on the identification of TrxR as an efficient biomarker to distinguish NSCLCs, benign lung diseases (BLDs), and healthy group, indicating its diagnostic value in NSCLC detection. In addition, this is the first study to compare the diagnostic value of TrxR activity with multiple NSCLC biomarkers, including NSE, CA19-9, CEA, and Cyfra21-1, and report the sensitivity and specificity of TrxR in combination with other NSCLCs biomarkers, which was distinct from the previous studies.

At present, CT scans have been a recommended approach of diagnosing NSCLC owing to their non-invasive nature. Despite this and other advantages, the contraindications for the use of this diagnostic approach including radiation exposure, high cost, and high false-positive rates must be considered. Compared to CT scans, TrxR activity detection is inexpensive, reproducible, easy (no need for extraction, purification, or reverse transcription) and convenient (requiring <2 ml plasma). Importantly TrxR activity also has a high sensitivity and specificity, making it a more competitive, efficient, effective, and reliable approach for diagnosing NSCLCs. The combination of Cyfra21-1, NSE, CEA, CA19-9, and TrxR, along with imaging and clinicopathologic examination information, may offer a better panel of diagnostic tools for the early and accurate diagnosis of NSCLCs, which will contribute to the prompt and effective treatment of NSCLCs and a reduction in associated mortality. However, as most participants in our study were recruited in central and eastern China, the presence of common confounding variables and comorbid conditions cannot be ignored. Given to the complex causes of NSCLCs and the variance in genomic and molecular signatures as a function of ethnicity, future studies using an independent cohort of patients will be needed. It will also be necessary to investigate the molecular mechanisms and clinical implications of elevated TrxR activity levels in cancer patients. In addition, in this study, we measured TrxR activity levels in plasma, and not in histologic specimen. It is still uncertain whether peripheral TrxR levels reflect similar changes in the corresponding tumor tissue, and so this topic also warrants further exploration.

In summary, this is the first large population study to report the clinically diagnostic relevance of TrxR activity as a plasma biomarker for NSCLCs in both training and validation cohorts. Our study showed that plasma TrxR activity is an effective and efficient biomarker of diagnosing NSCLCs, particularly as a means of characteristic discrimination among tumors of different metastatic states and histological differentiation. Hence, we recommend the convenient, economical, relatively non-invasive, and reproducible detection of TrxR activity levels, along with CEA, NSE, and Cyfra21-1, to aid in diagnosis of NSCLC.

## Supplementary information

Supplementary Info
