# Supplementary material for: Thioredoxin Reductase as a Novel and Efficient Plasma Biomarker for the Detection of Non-Small Cell Lung Cancer: a Large-scale, Multicenter study
Source: Sci Rep. 2019 Feb 25;9:2652. doi: 10.1038/s41598-018-38153-7 (PMC6389956; doi:10.1038/s41598-018-38153-7)

**Thioredoxin Reductase as a Novel and Efficient Plasma Biomarker for the Detection of Non-Small Cell Lung Cancer: a Large-scale, Multicenter study**

Suofu Ye^1, #^, Xiaofeng Chen^2, #^, Yi Yao^3, #, *^, Yueqin Li^4^, Ruoxuan Sun^1^, Huihui Zeng^1^, Yongqian Shu^2, *^, Hanwei Yin^4, *^

Authors Affiliations: ^1^State Key Laboratory of Natural and Biomimetic Drugs, Peking University Health Science Center, Beijing, China; ^2^Department of Oncology, the First Affiliated Hospital of Nanjing Medical University, Nanjing, China；^3^Cancer Center, Renmin Hospital of Wuhan University, Wuhan, China; ^4^Keaise Center for Clinical Laboratory, Wuhan, China

^#^ These authors contributed equally to this work

*Corresponding Author:

Hanwei Yin: Keaise Center for Clinical Laboratory, Wuhan 430075, China. E-mail address: 18911420767@163.com.

Yongqian Shu: Department of Oncology, the First Affiliated Hospital of Nanjing Medical University, Nanjing 210029, China. E-mail address: shuyongqian@csco.org.cn.

Yi Yao: Cancer Center, Renmin Hospital of Wuhan University, Wuhan 430060, China. E-mail address: yiyaowhu@qq.com

**Supplemental Experimental Procedures**

**Sample preparation for TrxR activity**

We followed a standard procedure to prepare the human specimens, which was also required by the manufacturer’s instruction. Samples from preoperative peripheral blood were collected in EDTA or anticoagulant-free tubes, followed by centrifugation at 3,000 rpm at room temperature for 5 minutes within 2 hours of collection. The supernatants were collected in 4°C and tested immediately. The storage temperature is 4°C, while the incubation temperature is 37°C. The low storage temperature and short handling time have made it least possible that the levels of free thiols in the plasma were altered during sample preparation or storage.

**Determination and calculation of TrxR activity**

As described in **Materials and methods**, TrxR activity was measured by commercially available thioredoxin reductase (TrxR) activity colorimetric assay kits (Bio Vision, Milpitas, CA, USA and Clairvoyance Health Technology Co., Ltd, Wuhan, China), which was based on DTNB reduction and performed according to the manufacturer’s instruction. Positive and negative controls from the kits are included in each reaction to monitor the assay performance.

The calculation of TrxR activity is described as follows:

**U/ml = ∆A412/min (thioredoxin reductase) × dil × Vol / (vol × L × ε)**

∆A412/min (thioredoxin reductase) = [∆A412/min (sample) - ∆A412/min (sample + inhibitor)]; dil = sample dilution factor; Vol = volume of reaction in ml; vol = volume of sample in ml; L = length of [optical path](http://www.baidu.com/link?url=qKlH69dtCI0DB-aFzmG_15ePnvC2oXnv-CuTmlytikvnActu4rnwBFBkjBZY2XjoO6dCnqVzRLU1C6xxM0nyXppWEbF8Uzhp5Tww-RU3BN5So2t61EGIn0F6dU6NznMD); ε = extinction coefficient; ∆A412/min (sample)= [A412 (sample, Xmin) - A412/min (sample,0min)] / Xmin; ∆A412/min (sample + inhibitor) = [A412 (sample + inhibitor, Xmin) - A412 (sample + inhibitor,0min)] / Xmin.

There are no significant difference of the levels and ranges of background absorbance between different patient groups. The average background absorbance [∆A412/min (sample + inhibitor)] is 0.113 for NSCLC, 0.111 for Benign Lung Disease (BLD), and 0.111 for Health Controls. The background absorbance of most patient samples ranges from 0.08-0.14.

The absolute values of absorbances of all patient samples were analyzed by KEA-TR100 analyzer (Clairvoyance, China), and its absolute absorbance linear range at 412 nm is 0.60-6.00. Any absolute absorbance outside this limitation was considered inaccurate and excluded from the study. Values of A412/min (sample,0min) and A412 (sample + inhibitor,0min) mostly ranges from 3.50-3.90, while those of A412 (sample, Xmin) and A412 (sample + inhibitor,Xmin) mostly ranges from 4.40-4.90 and 4.10-4.50, respectively.

**Supplemental Figure S1. Study profile.**


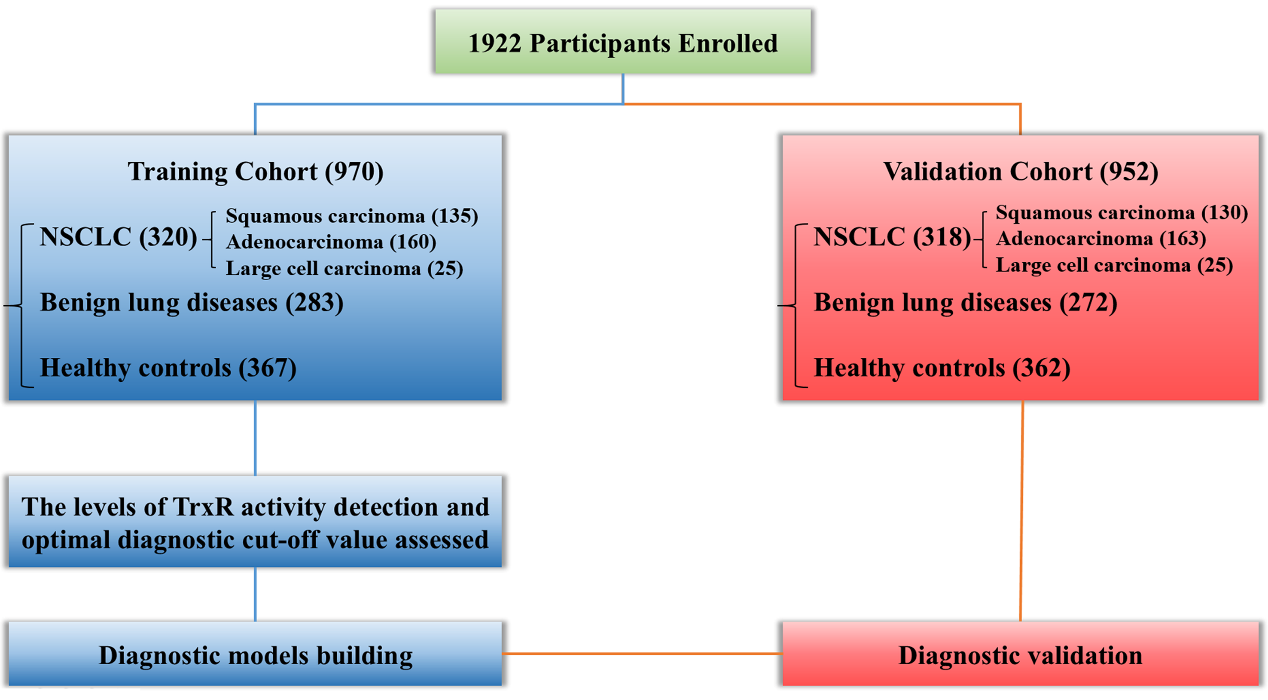

Supplement: Supplementary file 1 — Supplementary Info [file 41598_2018_38153_MOESM1_ESM.docx]
